# Benzofuroisoquinoline derivatives reverse amyloidogenesis and amyloid‐associated memory impairments in acute and transgenic mouse models of Alzheimer’s disease

**DOI:** 10.1002/alz.094924

**Published:** 2025-01-09

**Authors:** InWook Park, Sohui Park, Hanna Jeon, Kyeonghwan Kim, Soljee Yoon, Jisu Shin, Hye Yun Kim, Ikyon Kim, YoungSoo Kim

**Affiliations:** ^1^ Yonsei University, Incheon, Incheon Korea, Republic of (South)

## Abstract

**Background:**

Alzheimer’s disease (AD) is predominantly caused by the aggregation of amyloid‐β (Aβ) peptides into neurotoxic plaques. Current therapeutic approaches using monoclonal antibodies, like aducanumab and lecanemab, have shown promise in reversing Aβ aggregation. However, these treatments are expensive and challenging to administer. Small molecules offer a more accessible alternative but face specificity hurdles. Our study explores a library of hybrid compounds, integrating structures known to affect Aβ aggregation, to find potential small molecule therapeutics for AD.

**Method:**

We utilized a thioflavin T (ThT) fluorescence assay to measure the inhibitory effects of YIAD compounds on Aβ aggregation. Compounds were tested at varying concentrations with preformed Aβ aggregates. For analysis of Aβ species post‐inhibition, SDS‐PAGE coupled with photocrosslinking (PICUP) and silver staining were employed. In vivo efficacy was assessed using an Aβ‐infused mouse model and a 5XFAD transgenic mouse model, with cognitive function measured by a Y‐maze test and Aβ plaque burden by thioflavin S (ThS) staining.

**Result:**

ThT assays indicated that certain YIAD compounds significantly inhibited Aβ aggregation. SDS‐PAGE analyses confirmed changes in Aβ species distribution upon compound treatment. Aβ aggregate dissociation was demonstrated with ThT assays, indicating potential for plaque clearance. YIAD‐0405 and 0407 significantly improved spatial memory in the Y‐maze test and reduced plaque burden in 5XFAD mice, as evident from ThS staining.

**Conclusion:**

Our findings suggest that YIAD compounds, specifically YIAD‐0405 and 0407, effectively inhibit and dissociate Aβ aggregates in vitro and in vivo. These compounds improve cognitive function and decrease plaque burden, positioning them as promising candidates for oral AD therapeutics. Further studies will explore their mechanistic pathways and long‐term efficacy in treating Alzheimer’s disease.

